# Influence of mercury exposure on blood pressure, resting heart rate and heart rate variability in French Polynesians: a cross-sectional study

**DOI:** 10.1186/1476-069X-10-99

**Published:** 2011-11-13

**Authors:** Beatriz Valera, Éric Dewailly, Paul Poirier, Emilie Counil, Edouard Suhas

**Affiliations:** 1 Axe Santé des Populations et Environnement, Centre de Recherche du CHUQ, 2875 Boulevard Laurier, Québec, G1V 2M2, Canada; 2 Department of Social and Preventive Medicine, Laval University, 1050 avenue de la Médecine, Quebec, G1V 0A6, Canada; 3 Quebec Heart and Lung Institute, Laval Hospital Research Centre, 2725 Chemin Sainte-Foy, Québec, G1V 4G5, Canada; 4 Faculty of Pharmacy, Laval University, 1050 avenue de la Médecine, Quebec, G1V 0A6, Canada; 5 Département Épidémiologie et Biostatistiques, École des Hautes Études en Santé Publique, Hôtel-Dieu, 1, place Notre-Dame, Paris Cedex 4, 75181, France; 6 Université Paris 13, GISCOP93, UFR SMBH, 74, rue Marcel Cachin, Bobigny Cedex, 93017, France; 7 Unité de maladies non transmissibles (LMNT), Institut Louis Malardé, rue du 5 mars 1797, Papeete, 98713, Polynésie Française

**Keywords:** Methymercury, blood pressure, heart rate variability, resting heart rate, n-3 fatty acids, French Polynesia

## Abstract

**Background:**

Populations which diet is rich in seafood are highly exposed to contaminants such as mercury, which could affect cardiovascular risk factors

**Objective:**

To assess the associations between mercury and blood pressure (BP), resting heart rate (HR) and HR variability (HRV) among French Polynesians

**Methods:**

Data were collected among 180 adults (≥ 18 years) and 101 teenagers (12-17 years). HRV was measured using a two-hour ambulatory electrocardiogram (Holter) and BP was measured using a standardized protocol. The association between mercury and HRV and BP parameters was studied using analysis of variance (ANOVA) and analysis of covariance (ANCOVA)

**Results:**

Among teenagers, the high frequency (HF) decreased between the 2^nd ^and 3^rd ^tertile (380 vs. 204 ms^2^, p = 0.03) and a similar pattern was observed for the square root of the mean squared differences of successive R-R intervals (rMSSD) (43 vs. 30 ms, p = 0.005) after adjusting for confounders. In addition, the ratio low/high frequency (LF/HF) increased between the 2^nd ^and 3^rd ^tertile (2.3 vs. 3.0, p = 0.04). Among adults, the standard deviation of R-R intervals (SDNN) tended to decrease between the 1^st ^and 2^nd ^tertile (84 vs. 75 ms, p = 0.069) after adjusting for confounders. Furthermore, diastolic BP tended to increase between the 2^nd ^and 3^rd ^tertile (86 vs. 91 mm Hg, p = 0.09). No significant difference was observed in resting HR or pulse pressure (PP)

**Conclusions:**

Mercury was associated with decreased HRV among French Polynesian teenagers while no significant association was observed with resting HR, BP, or PP among teenagers or adults

## Background

Seafood may content high levels of mercury since this contaminant is transformed into methylmercury (MeHg) in the aquatic environment and it is accumulated in predator fish and marine mammals [1]. In French Polynesia, data collected among adults from Tahiti and Moorea Islands revealed high blood mercury concentrations (90.3 nmol/L) [2] compared to populations with less seafood consumption like Canada (4.6 nmol/L), Germany (2.9 nmol/L) or the United States (4.2 nmol/L) [3]. In addition, cord blood samples collected among 241 delivering women from all French Polynesian islands revealed a mean mercury concentration of 13 μg/L (65 nmol/L) and, 82.5% of the samples had levels above the US-EPA blood guidelines (5.8 μg/L or 29 nmol/L) [4].

Epidemiological and experimental evidence suggests a negative impact of chronic mercury exposure on the cardiovascular system. Chronic mercury exposure has been associated with increased risk of myocardial infarction, progression of atherosclerosis and oxidative stress (see review by Roman et al.) [5]. Furthermore, exposure to this contaminant has been associated with increased blood pressure (BP) and decreased heart rate variability (HRV) among children and adults. HRV reflects the cardiac parasympathetic and sympathetic activities of the autonomic nervous system (ANS) and, reduced HRV can lead to sudden cardiac death (SCD) [6]. In the Faeroe Islands, prenatal mercury exposure was associated with decreased HRV at age seven and fourteen years old [7,8]. A deleterious impact of prenatal mercury exposure on HRV was also reported by Oka et al. [9] and Murata et al. [10] among Japanese children with high prenatal mercury exposure. Regarding adults, a negative impact of mercury on HRV parameters was observed during an intervention study conducted in Japan where the experimental group followed a diet rich in tuna or swordfish during 14 weeks [11]. A negative association between mercury and HRV was also observed in a cross-sectional study conducted among residents living near an industrial area in Korea [12]. In addition, our research team has observed a negative impact of mercury on HRV in native populations from Canada such as the Inuit from Nunavik [13]. Regarding BP, prenatal mercury exposure was associated with increasing systolic blood pressure (SBP) at seven years old in the Faeroe Islands [7] but this effect was not discernible at 14 years old [8]. Furthermore, a positive association between prenatal mercury exposure and diastolic blood pressure (DBP) at 15 years old was observed among boys in the Seychelles Islands [14]. Among adults, mercury exposure has been associated with increased BP in populations like the Inuit from Nunavik [15], Faroese whaling men [16] and inhabitants of the Brazilian Amazonian [17]. Furthermore, mercury was associated with decreasing DBP and increased pulse pressure (PP) among Greenlanders [18].

Considering that French Polynesians are exposed to mercury through the diet and that this contaminant could affect cardiovascular risk factors such as BP and HRV, we aimed to assess the association between mercury and these physiological parameters considering fish nutrients [n-3 polyunsaturated fatty acids (n-3 PUFAs) and selenium] and other potential confounding factors in two distinct groups of Polynesians; the first one living in a large city (Papeete) and, the second in a rural and remote island of the Austral archipelago.

## Methods

### Study population and sampling

**Figure 1:**
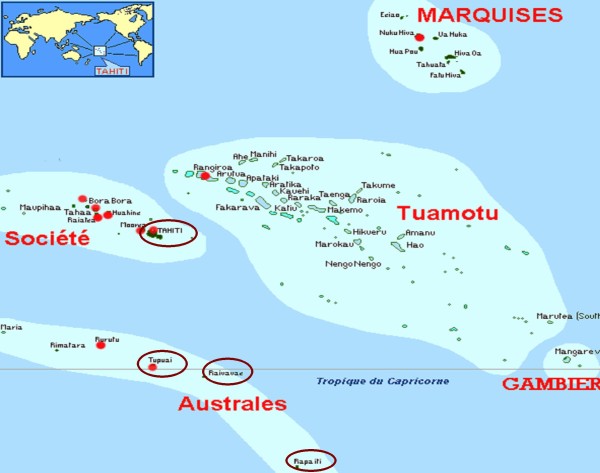
**Map of French Polynesia**. Tupuai is equivalent to Tubuai. Papeete is the capital of Tahiti.

The health survey "Dietary and epidemiologic transition in French Polynesia" was conducted among adults and teenagers in French Polynesia in 2007. To insure genetic comparability, the adult sample (≥ 18 years old) included adults born in the Austral Islands but living in Papeete (urban lifestyle) and adults born in the Austral Islands and living in Tubuai (rural lifestyle) (Figure 1). A random sampling stratified on age (18-49 and ≥ 50 years) and gender (target: 50 participants per age group per island) was carried out using the electoral lists from Papeete and Tubuai that had been updated in December 31^th ^2006. However, in Papeete, subjects randomly selected were difficult to contact and it was necessary to use a snowball procedure. The latter consist of asking the participants to nominate other individuals who could be contacted to participate in the study. In Papeete, 493 adults were enrolled in the electoral list, 109 eligible subjects were contacted and 87 accepted to participate. Of the 1349 adults enrolled on the electoral list of Tubuai, 305 were contacted while 102 were eligible and consented to participate.

We also recruited teenagers (12-17 years old) born in the Austral Islands but living in Papeete and, born in the Austral Islands and living in the islands of Raivavae, Rapa and Tubuai (Figure 1). A random sampling stratified on gender was carried out for each island (target: 30 participants per island subgroup) using the student list from two colleges in Tubuai that included students from the three islands. From 326 teenagers enrolled in one of the two colleges in fall 2006, 114 were contacted and 89 were eligible and accepted to participate. In Papeete, we asked adult participants if it would be possible to interview their children. This strategy was followed since it was not possible to identify *a priori *children born in the Austral Islands. The sample was completed through a second study wave conducted in a secondary school in the Austral district of Papeete. In total, the sample included 28 teenagers from Papeete, 30 from Raivavae, 30 from Rapa and 29 from Tubuai. All participants signed an informed consent and the research protocol was approved by the Ethic committee of French Polynesia.

### Data collection

#### Questionnaires

Questionnaires from the STEPS instrument (WHO STEPwise approach to surveillance) [19,20] were adapted to the Maohi context and used to collect information regarding age, gender, alcohol consumption, smoking habits and anti-hypertensive treatment.

#### Anthropometric measurements

The height of participants was obtained using a rigid squared and measuring tape, as they stood barefoot on a hard surface with their back against a wall. Waist circumference (WC) was measured after exhalation with the tape placed horizontally to where the abdomen curves in. If a subject's waist was not sufficiently defined, he or she was measured at roughly the location of the last floating rib [21]. All of the foregoing measurements were recorded to the nearest centimetre. Body mass index (BMI) was calculated as weight (kg)/height (m)^2^.

#### Heart rate variability (HRV), blood pressure (BP) and resting heart rate (HR)

HRV indices were derived from a two-hour Holter ambulatory monitoring system (GE MARQUETTE SERIE 8500) with a recording frequency of 128 Hz. Seven leads (derivations V5, V1, and AVF) were installed when subjects arrived to the clinic and we obtained a recording of HR during to two hours. Interpretation and extraction of HRV parameters were performed automatically by using the software provided by General Electric (MARS PC Ambulatory ECG Analysis System) while complete signal was carefully edited using visual checks and manual corrections of individual R-R intervals and QRS complex classifications. For the calculation of the R-R parameters, only R-R intervals between QRS complexes of sinusal origin were used. Intervals whose duration was < 80% or > 120% of that of the running R-R average were excluded. Time domain parameters included the standard deviation of R-R intervals (SDNN), the standard deviation of the average R-R intervals calculated over five-minute periods (SDANN) and the square root of the mean squared differences of successive R-R intervals (rMSSD). SDNN and SDANN represent the overall HRV while rMSSD is an index of parasympathetic activity. Fast Fourier transformation was used to compute frequency domain parameters such as low frequency (LF = 0.04-0.15 Hz), which represents both sympathetic and parasympathetic activity, and high frequency (HF = 0.15-0.40 Hz), which is a specific index of parasympathetic activity. The LF/HF ratio represents the sympatho-vagal balance [22]. LF and HF were also expressed in normalised units.

BP was measured according to a standardized protocol using mercury sphygmomanometers, 15-inch stethoscopes, and cuffs sized to the subjects' arms [23]. Prior to having their blood pressure taken, subjects had rested for five minutes and not eaten or smoked for at least thirty minutes. Each subject had three BP readings and means of systolic blood pressure (SBP) and diastolic blood pressure (DBP) were calculated using the last two readings. Pulse pressure (PP) was calculated as the difference between SBP and DBP. Resting HR was measured by taking the pulse in the right wrist for 30 seconds or 60 seconds if the pulse was irregular.

#### Laboratory analyses

Mercury and selenium were determined in blood which is a biomarker of recent exposure [24]. Determination of mercury and selenium was performed by inductively coupled plasma mass spectrometry (ICP-MS). The analysis of selenium was performed in whole blood. For mercury determination, samples were diluted 20-fold in a solution containing ammonium hydroxide before analysis. The detection limit for mercury and selenium was 0.5 nmol/L and 0.1 μmol/L respectively, and each run of samples included a standard. The inter-assay variability for the mercury and selenium measurements was 2.1% and 6.1% respectively. Analyses were performed by the INSPQ Human Toxicology Laboratory, which is accredited ISO 17025 by the Standards Council of Canada. This laboratory is also an international leader in analytical toxicology applied to human and environmental studies and a reference institution for interlaboratory comparison programs in heavy metals measurements. Concentrations of triglycerides, low-density lipoprotein cholesterol (LDL-cholesterol) and high-density lipoprotein cholesterol (HDL-cholesterol) were determined according to methods of the Lipid Research Clinics (US Department of Health). Determination of insulin was performed with a Roche Modular analytics E170 (Elecsys module) auto-analyzer using a commercial double-antibody radioimmunoassay.

The fatty acid composition of the erythrocyte membranes was measured after membrane purification, chloroform/methanol lipid extraction and methylation of fatty acids, followed by capillary gas-liquid on a HP5890 gas chromatograph (Hewlett Packard, Toronto, ON) equipped with a HP8823 capillary column coupled with a flame ionization detector (FID). Results were expressed as percent of total fatty acids.

### Statistical analyses

A descriptive analysis was conducted for all variables. Normality was examined using graphic plots and the Shapiro-Wilk test and skewed variables were log-transformed. Arithmetic means and standard deviation (SD) were calculated for normally distributed variables while geometric means and 95% confidence interval are presented for log-transformed variables. Median (25^th ^and 75^th ^percentiles) are presented for all continuous variables. Proportions were calculated for categorical variables. Socio-demographic and clinical variables were compared between sexes and communities using the t-student test or the analysis of variance (ANOVA) for continuous variables while the chi-squared test was used for categorical variables. The simple relationship between mercury and BP and HRV was studied using analysis of variance while the analysis of covariance (ANCOVA) was applied in order to adjust for confounders. Analyses were conducted separately among adults and teenagers. Variables considered as potential confounders were: age, sex, fasting glucose, triglycerides, WC, total n-3 PUFAs, selenium and anti-hypertensive treatment. Among teenagers, models were not adjusted for the anti-hypertensive treatment since none was taking this kind of medication. We also verified the statistical interaction between mercury and sex, total n-3 PUFAs and selenium. Two tailed tests were conducted and a p-value < 0.05 was considered statistically significant. The analyses were carried out using the SAS software (version 9.1; SAS Institute Inc., Cary, NC, USA).

## Results

From 189 adults who participated in the study, three were excluded because blood mercury could not be determined, six had missing data regarding BP and 15 regarding HRV. After excluding subjects with missing values on some adjustment variables, multivariable analyses on BP were conducted among 157 adults while multivariable analyses on HRV were restricted to 146 adults. Regarding teenagers, from 117 who participated in the study, two were excluded due to missing data on mercury, 14 did not have data on BP and 19 on HRV. After excluding participants with missing values on some adjustment variables, the final sample for multivariable BP analyses involved 82 teenagers while HRV multivariable analyses were conducted among 78 teenagers.

The descriptive results for all variables are presented in Table 1 and Table 2. Blood mercury levels were higher in men than women (17.8 vs. 11.1 μg/L respectively, p < 0.0001). Fifty three adults (29.4%) had blood mercury levels in the category of "increased risk" (20-100 μg/L or 100-500 nmol/L) while three (1.7%) were considered "at risk" (≥ 100 μg/L or 500 nmol/L) according to Health Canada recommendations. In addition, five teenagers (4.9%) had mercury levels in the category of "increased risk". Ninety-seven subjects (53.9%) were classified as hypertensive (SBP ≥ 140 mm Hg or DBP ≥ 90 mm Hg or antihypertensive medication). Significant difference was observed between men and women regarding the lipid profile; men presented higher levels of LDL-cholesterol (3.43 vs. 3.07 mmol/L, p = 0.02) and lower levels of HDL-cholesterol (1.22 vs. 1.36 mmol/L, p = 0.004) while levels of triglycerides did not differ between women and men (p = 0.09). Furthermore, men presented higher WC (174 vs. 163 cm, p < 0.0001) and higher levels of DHA (6.69 vs. 6.17% of total fatty acids, p = 0.02), EPA (0.91 vs. 0.66% of total fatty acids, p < 0.0001), lead (29.4 vs. 18.3 μg/L, p < 0.0001) and selenium (288.2 vs. 235.7 μg/L, p = 0.0003). Regarding teenagers, most of the characteristics were similar in boys and girls except for triglycerides and BMI that were higher in girls than boys (1.04 vs. 0.77 mmol/L, p = 0.003 and 25.6 vs. 23.7 Kg/m^2^, p = 0.04; respectively), and WC, fasting glucose and blood lead that were lower in girls than boys (164 vs. 171 cm, p < 0.0001, 4.99 vs. 5.27 mmol/L, p = 0.0003 and 18.4 vs. 24.5 μg/L, p = 0.001; respectively). Furthermore, the comparison of socio-demographic and clinical variables between communities showed higher levels of mercury (19.7 vs. 11.1 μg/L, p < 0.0001), total n-3 PUFAs (9.7 vs. 8.7% of total fatty acids, p = 0.006) and, selenium (290 vs. 235 μg/L, p = 0.0002) among adults from Papeete. Similarly, the proportion of drinkers (51 vs. 39%, p = 0.006) and smokers (55 vs. 47%; p = 0.02) was higher in Papeete. Among teenagers, significant differences were observed regarding mercury levels, which were higher in Rapa (9.7 μg/L) and Raivavae (10.9 μg/L) compared to Papeete (5.9 μg/L) (p < 0.05). In addition, LDL-cholesterol was lower in Rapa compared to Tubuai (1.9 vs. 2.4 mmol/L, p < 0.05) while the lowest levels of fasting glucose were observed in Papetee compared to Tubuai (4.6 vs. 5.2 mmol/L) and Rapa (5.2 mmol/L, p < 0.05). Furthermore, LF (555 vs. 1064 ms^2^, p < 0.05), HF (196 vs. 639 ms^2^, p < 0.05) and rMSSD (31 vs. 46 ms, p < 0.05) were lower in Raivavae compared to Papeete while no significant difference was observed with respect to other communities.

**Table 1:** Characteristics of the participants

Variables	Teenagers (12-17 years old) (n = 101)			Adults (≥ 18 years old) (n = 180)		
	
	Mean (SD or 95% CI)	Median (25^th^-75^th ^percentiles)	Proportion (%)	Mean (SD or 95% CI)	Median (25^th ^-75^th ^percentiles)	Proportion (%)
Age (years)	14.2 (1.5)	14.0 (13.0-15.0)		48.6 (14.8)	48.0 (39.0-58.0)	

Gender (males, %)			44 (43.6%)			85 (47.2%)

Mercury (μg/L)*	8.1 (7.2-9.1)	8.5 (6.3-11.0)		14.5 (12.9-16.4)	13.5 (8.5-22.0)	

LDL-cholesterol (mmol/L)	2.15 (0.62)	2.09 (1.71-2.50)		3.25 (0.94)	3.25 (2.62-3.81)	

HDL-cholesterol (mmol/L)	1.19 (0.24)	1.16 (1.03-1.35)		1.29 (0.29)	1.24 (1.06-1.56)	

Triglycerides (mmol/L)*	0.90 (0.82-1.00)	0.86 (0.66-1.14)		1.23 (1.14-1.34)	1.15 (0.82-1.82)	

BMI (kg/m^2^)	24.9 (5.6)	23.9 (21.2-27.1)		32.7 (7.9)	31.1 (27.3-36.5)	

Waist circumference (cm)	165.0 (15)	165.5 (161.0-172)		168.2 (9.3)	168.0 (161.0-175.0)	

Fasting glucose (mmol/L)*	5.1 (5.0-5.2)	5.2 (4.9-5.4)		5.9 (5.6-6.1)	5.5 (5.0-6.3)	

Fasting insulin (pmol/L)*	96.9 (85.5-110.1)	97.0 (69.0-140.0)		64.9 (57.9-72.7)	69.0 (38.0-101.0)	

EPA (% of total fatty acids)*	0.43 (0.41-0.45)	0.42 (0.36-0.49)		0.77 (0.72-0.83)	0.71 (0.54-1.08)	

DHA (% of total fatty acids)	4.49 (0.93)	4.50 (3.87-5.12)		6.41 (1.38)	6.22 (5.56-7.26)	

Total n-3 PUFAs (% of total fatty acids)	6.79 (0.99)	6.72 (6.12-7.51)		9.24 (1.86)	8.97 (8.00-10.24)	

Selenium (μg/L)*	165.2 (159.5-171.2)	170.0 (150.0-190.0)		259.9 (245.8-274.9)	250.0 (200.0-320.0)	

Lead (μg/L)*	20.9 (19.1-22.8)	21.0 (15.0-29.0)		23.1 (21.4-24.9)	21.5 (16.0-29.0)	

Smoking habits (yes, %)			26 (25.7%)			102 (56.7%)

Alcohol consumption (yes, %)			33 (33.3%)			90 (51.1%)

Anti-hypertensive treatment (yes, %)			0			41 (22.8%)

* For variables not normally distributed, the geometric means and 95% confidence interval (CI) are presented; SD: Standard deviation; μg/L can be converted nmol/L multiplying by 5.

**Table 2:** Blood pressure (BP), resting heart rate (HR) and heart rate variability (HRV) among adults and teenagers

Dependent variables	Teenagers (12-17 years old)		Adults (≥ 18 years old)	
	
	Mean (SD or 95% CI)	Median (25^th^-75^th ^percentiles)	Mean (SD or 95% CI)	Median (25^th ^-75^th ^percentiles)
SBP (mm Hg)	111 (14)	110 (100-120)	133 (22)	130 (120-140)

DBP (mm Hg)	74 (11)	70 (65-80)	84 (15)	81 (75-90)

PP (mm Hg)	38 (12)	39 (30-45)	49 (16)	48 (40-58)

Resting HR (bpm)	74 (12)	72 (66-84)	67 (11)	66 (60-72)

LF (ms^2^)*	771 (674-883)	711 (523-1250)	374 (312-447)	435 (211-824)

LF norm (nu)	70.6 (9.0)	70.6 (63.8-77.7)	72.7 (11.9)	74.7 (66.7-81.5)

HF (ms^2^)*	303 (251-367)	338 (162-641)	129 (108-154)	138 (75-262)

HF norm (nu)	29.4 (9.0)	29.4 (22.3-36.2)	27.3 (11.9)	25.3 (18.5-33.3)

LF/HF*	2.54 (2.32-2.79)	2.40 (1.76-3.48)	2.89 (2.61-3.22)	2.95 (2.00-4.41)

SDNN (ms)*	89 (84-95)	88 (71-108)	80 (76-84)	83 (67-102)

SDANN (ms)*	56 (52-60)	56 (44-72)	53 (50-56)	56 (42-71)

rMSSD (ms)*	36 (33-39)	36 (27-49)	29 (27-31)	28 (21-39)

* For variables not normally distributed, the geometric means and 95% confidence interval (CI) are presented; SD: standard deviation.

Among adults, blood mercury levels increased with age (r = 0.16, p = 0.035) and was strongly correlated with selenium (r = 0.67, p < 0.0001), as well as more modestly with DHA (r = 0.52, p < 0.0001), EPA (r = 0.37, p < 0.0001) and total n-3 PUFAs (r = 0.48, p < 0.0001). Among teenagers, significant correlations were also observed between blood mercury and selenium (r = 0.45, p < 0.0001), DHA (r = 0.39, p < 0.0001), EPA (r = 0.44, p < 0.0001) and total n-3 PUFAs (r = 0.39, p < 0.0001). The interaction terms between mercury and sex, DHA, EPA, total n-3 PUFAs and selenium were not statistically significant in adults and teenagers (p > 0.05).

**Figure 2:**
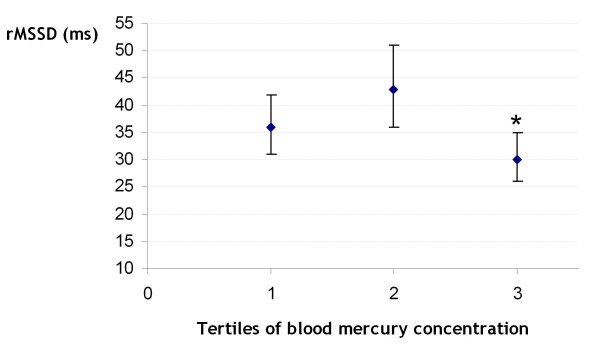
**Adjusted rMSSD means across tertiles of blood mercury concentrations among teenagers**. * p _diff 2-3 _= 0.004. Geometric means ± SD are presented. Means were adjusted for age, sex, waist circumference, fasting glucose, triglycerides, selenium and total n-3 PUFAs.

The results of the ANCOVA analyses among teenagers showed a significant decrease in HF and rMSSD between the 2^nd ^and 3^rd ^tertile after adjusting for confounders (Figure 2). In addition, LF/HF increased between the 2^nd ^and 3^rd ^tertile and the difference between the 1^st ^and 3^rd ^tertile was near the significance level. However, means of SBP, DBP, PP and resting HR did not vary significantly across tertiles of blood mercury concentration (Table 3). Among adults, significant differences were not observed in HRV parameters after adjusting for confounders (Table 4). Regarding BP, increases in SBP and DBP were observed but the differences did not reach the significant level. In contrast, no significant difference was observed in PP and resting HR means across tertiles of blood mercury concentration (Table 4). Similar results were obtained when analyses were restricted to subjects without anti-hypertensive medication.

**Table 3:** Results of ANOVA and ANCOVA analyses among teenagers

	T1: 1.2-7.3	T2: 7.4-10.0	T3: 11.0-26.0	
**BP and resting HR**	**Adjusted means ± SEM ^&^**			**p trend**

SBP (mm Hg)	109 ± 2.9	112 ± 2.8	113 ± 2.9	0.38

DBP (mm Hg)	71 ± 2.4	72 ± 2.3	74 ± 2.3	0.49

PP (mm Hg)	37 ± 2.2	40 ± 2.1	38 ± 2.2	0.66

Resting HR (bpm)	77 ± 2.6	72 ± 2.5	74 ± 2.6	0.47

	**T1: 1.2-7.4**	**T2: 7.9-10.0**	**T3: 11.0-26.0**	

**HRV variables**	**Adjusted means ± SEM ^&^**			

LF (ms^2^)*	788 ± 1.2	863 ± 1.2	614 ± 1.2	0.21

LF norm (nu)*	69 ± 1.9	69 ± 1.9	74 ± 1.8 ^a^	0.08

HF (ms^2^)*	344 ± 1.2	380 ± 1.2	204 ± 1.2 ^a^	0.07

HF norm (nu)*	31 ± 1.9	31 ± 1.9	26 ± 1.8	0.08

LF/HF*	2.3 ± 1.1	2.3 ± 1.1	3.0 ± 1.1 ^a^	0.08

SDNN (ms)*	89 ± 1.1	92 ± 1.1	82 ± 1.1	0.44

SDANN (ms)*	54 ± 1.1	57 ± 1.1	52 ± 1.1	0.77

rMSSD (ms)*	37 ± 1.1	43 ± 1.1	30 ± 1.1^a^	0.11

SEM: standard error of the mean. * Log-transformed variables. ^&^Models were adjusted for age, sex, waist circumference, fasting glucose, triglycerides, selenium and total n-3 PUFAs. Letters indicate significant differences at p < 0.05. ^a ^Difference between T2 and T3.

**Table 4:** Results of ANOVA and ANCOVA analyses among adults

	T1: 1.4-10.0	T2: 11.0-18.0	T3: 19.0-111.0	
**BP and resting HR**	**Adjusted means ± SEM ^&^**			**p-trend**

SBP (mm Hg)	137 ± 3.2	136 ± 3.1	140 ± 3.2	0.48

DBP (mm Hg)	86 ± 2.3	86 ± 2.3	91 ± 2.3	0.13

PP (mm Hg)	51 ± 2.3	50 ± 2.2	49 ± 2.2	0.57

Resting HR (bpm)	66 ± 1.7	69 ± 1.7	66 ± 1.7	0.82

	**T1: 1.4-9.8**	**T2: 10.0-18.0**	**T3: 19.0-111.0**	

**HRV variables**	**Adjusted means ± SEM ^&^**			

LF (ms^2^)*	403 ± 1.2	314 ± 1.2	403 ± 1.2	0.98

LF norm (nu)*	72 ± 1.9	70 ± 1.9	74 ± 2.0	0.50

HF (ms^2^)*	145 ± 1.2	122 ± 1.2	132 ± 1.2	0.66

HF norm (nu)*	28 ± 1.9	30 ± 1.9	26 ± 2.0	0.50

LF/HF*	2.8 ± 1.1	2.6 ± 1.1	3.2 ± 1.1	0.47

SDNN (ms)*	84 ± 1.1	75 ± 1.1	82 ± 1.1	0.77

SDANN (ms)*	56 ± 1.1	49 ± 1.1	54 ± 1.1	0.68

rMSSD (ms)*	31 ± 1.1	27 ± 1.1	30 ± 1.1	0.66

SEM: standard error of the mean. * Log-transformed variables. ^&^Models adjusted for age, sex, waist circumference, fasting glucose, triglycerides, anti-hypertensive treatment, selenium and total n-3 PUFAs.

## Discussion

French Polynesians consume high quantities of fish, which are rich in nutrients such as n-3 PUFAs and selenium, but that may also content high mercury levels. In the present study, mercury exposure was associated with decreased HRV parameters (HF and rMSSD) among teenagers after adjusting for fish nutrients and HRV risk factors. These results suggest a negative impact on the parasympathetic activity of the ANS. Among adults, some HRV parameters decreased across tertiles of blood mercury concentrations but the differences only approached the significance level. Furthermore, no significant difference was observed for resting HR, PP or BP across tertiles of blood mercury concentrations among adults and teenagers.

A negative impact of mercury on HRV has been observed among Inuit adults from Nunavik [13], among residents near an industrial area in Korea [12] and in an intervention study conducted in Japan [11]. Our results partially agree with previous studies since we only observed significant associations among teenagers although the differences were near the significant level among adults. With respect to the Inuit from Nunavik [13], levels of mercury and n-3 PUFAs were similar in both populations. However, the French Polynesian diet is rich in antioxidants, which could protect this population from the impact of mercury [25,26]. Antioxidants such as glutathione and L-cystein could bind mercury and increase its excretion [27]. With respect to the study conducted in Korea [12], mercury exposure was higher among Polynesians. However, the smaller sample size used in our study could reduce the statistical power and decrease the probabilities of detecting significant associations. Our results are not in accordance with those observed in an intervention study conducted in Japan [11]. However, mercury levels were higher in the experimental group (35 μg/L or 175 nmol/L) than in our study, which could explain the differences in results. Finally, the results observed among teenagers are in line with a previous study conducted in Faroe Islands [8] showing a negative impact of mercury exposure during childhood on HRV.

Our results regarding resting HR are in accordance with those obtained in a study conducted among Faroese whaling men [16] and, in an intervention study conducted in Japan [11] where no significant effect of mercury has been observed. In contrast, in a case-control study involving patients with foetal Minamata disease (FMD), Oka et al., observed a negative association between mercury and mean R-R, which is the inverse of the HR [9]. However, mercury exposure in FMD patients was higher than in our sample [28]. Furthermore, the results observed among teenagers agree with a previous study conducted in Faroe Islands [8] in which no significant association was observed between mercury exposure during childhood and HR.

A negative impact of mercury exposure on BP has been mainly reported in populations with high seafood consumption [15-18]. The results of the present study are not in accordance with previous studies since we did not observed significant differences across tertiles of blood mercury concentration. Among adults, SBP and DBP means increased between the 2^nd ^and 3^rd ^tertile but the relatively small sample size could have decreased the statistical power and the likelihood of detecting significant differences. Furthermore, differences in exposure levels and dietary habits could also explain the discrepancies with respect to previous studies. With respect to the Inuit from Nunavik [15], mercury levels are comparable in both populations. However, French Polynesians may be protected by antioxidants present in tropical fruits and vegetables [25]. A diet rich in antioxidants can improve endothelial function among volunteers at low cardiovascular risk [29]. Our results also disagree with those obtained by Pedersen et al. among Greenlanders [18]. That population presents high mercury levels but also high n-3 PUFAs levels [30] which could influence BP [31]. However, statistical models were only adjusted for age and BMI, which could result in some residual confounding bias. A positive association between blood mercury and SBP and DBP was also observed among Faroese whaling men [16] but mercury levels (29.5 μg/L or 147.5 nmol/L) were higher than in our sample. Our results also disagree with those obtained among Brazilian Amazonian residents [17] but hair mercury levels were higher than in our sample (mean: 17.8 μg/g equivalent to 71.2 μg/L or 356 nmol/L in blood), which could explain the discrepancies in results. In addition, blood mercury was associated with increased SBP among women who participated in the NHANES 1999-2000; significant associations were only observed among non-fish consumers [32]. Fish nutrients could have played a protective role among fish consumers but these substances were not included in the analyses. In addition, the sample size is higher than in our study, which increases the statistical power. The results obtained among teenagers are in accordance with previous studies conducted in Faeroe Islands [8] where exposure during childhood was not associated with increasing BP.

A limitation of the present study concerns the different sampling methods used in Papeete and the Austral Islands, which limits the generalization of the results. In Papeete, it was necessary to use the snowball procedure instead of a simple random sampling since adults randomly selected were difficult to identify and contact. The information regarding the native island does not appear with precision in the electoral list. Consequently, adults born in the Austral Islands and living in Papeete could be identified only by using the information provided by their parents. However, despite this limitation, the results of the present study are pertinent for this population since no information was available regarding the relationship between contaminants and cardiovascular risk factors. Another limitation concerns the use of resting BP measurements instead of ambulatory BP. Although the method used in this study is in agreement with the European [33] and American guidelines [34], ambulatory BP would permit to obtain more BP measurements and thus, better reflect the real BP. In resting conditions, the parasympathetic activity is higher than the sympathetic activity, which leads to lower BP values than those obtained during the daytime in ambulatory conditions. It is possible that the fact of taking resting BP measurements underestimates the mercury effect. However, Pedersen et al. [18] obtained similar associations between blood mercury and DBP and PP measured during daytime and during 24 h even in presence of higher BP values during the daytime period. Moreover, it should be considered that ambulatory BP measurements may be difficult to be performed during health surveys conducted in remote regions as is the case of French Polynesia.

Among the strengths of this study, it is of note the minimization of the information and confounding bias. First of all, mercury determination was carried out by the INSPQ Human Toxicology Laboratory, which is accredited ISO 17025 by the Standards Council of Canada. Secondly, the method used for recording HRV is placebo-free since it permits to measure HRV during routine activities [35]. Regarding the confounding bias, we tried to consider most HRV and BP risk factors (age, gender, triglycerides, fasting glucose and obesity). Smoking and alcohol consumption were not included in the models since no significant association was observed with HRV and BP in our sample. However, we were not able to adjust for sodium intake or physical activity which could have influenced the results. In one hand, sodium intake was estimated using a 24 h recall questionnaire and the high variability intra-subject does not allow using these data as an individual estimate of sodium intake. On the other hand, physical activity could not be accurately measured since most of the participants did not return the pedometers. In addition to traditional BP and HRV risk factors, we also took into account the impact of fish nutrients (selenium and n-3 PUFAs), which are related to mercury and could affect BP or HRV [31,36]. No adjustment for these substances could lead to residual confounding and thus, control of these substances is imperative in populations where mercury exposure is mainly due to fish consumption.

## Conclusions

Mercury was associated with decreased HRV among French Polynesian teenagers. However, no significant association was observed with resting HR, BP, or PP among teenagers or adults.

## List of abbreviations

HRV: heart rate variability; SDNN: standard deviation of R-R intervals; SDANN: standard deviation of the average R-R intervals calculated over 5-minute periods; rMSSD: square root of the mean squared differences of successive R-R intervals; LF: low frequency; HF: high frequency; SBP: systolic blood pressure; DBP: diastolic blood pressure; PP: pulse pressure; WC: waist circumference; BMI: body mass index; PUFAs: polyunsaturated fatty acids; DHA: Docosahexaenoic acid; EPA: Eicosapentaenoic acid; ANS: autonomic nervous system; SCD: sudden cardiac death; MeHg: methylmercury; FMD: foetal Minamata disease.

## Competing interests

The authors declare that they have no competing interests.

## Authors' contributions

BV performed the statistical analyses, participated in the interpretation of the results and drafted the manuscript. ED, EC and ES conceived the study, participated in its design and in the interpretation of the results. PP participated in the interpretation of the results. All authors read and approved the final manuscript.
